# Supplementary material for: Women’s experiences of anal incontinence following vaginal birth: A qualitative study of missed opportunities in routine care contacts
Source: PLoS One. 2023 Jun 27;18(6):e0287779. doi: 10.1371/journal.pone.0287779 (PMC10298771; doi:10.1371/journal.pone.0287779)
Supplement: S1 Table — (DOCX) [file pone.0287779.s001.docx]

S1 Table of additional quotes

These quotes are in addition to quotes included in the main text of the manuscript and further describe women’s experiences in line with the themes and sub-themes identified.

| Theme | Quote and reference |
| --- | --- |
| 1. Opportunities for diagnosis missed  Normalisation | *So I suppose it’s almost, you know, my assumption was that it happened quite a lot and loads of people have it. Yeah, it’s just another thing that women have to put up with (P12, menopause, 45-54).* |
| 2. Missed opportunities for information sharing  Lack of debrief or unanswered questions after birth    **Lack of awareness of AI**  **Linking to information opportunities antenatally** | *Still, probably probably feel like I still, I don’t know, probably still not understanding 100%. Obviously I said that I had conflicting advice still feel as though even afterwards I feel like the only person who was more kind of aware was the women’s physio. But again, it kinda it’s, you kinda feel like you just on your own with it I guess (P11, post-natal, 25-34).*  *But even even when I was discharged from hospital, there was no follow up about, well, you know, you need to maybe take some lactulose or something that’s gonna help, or at least ease going to the toilet for a few weeks while while you, while it does all heal up. It was just a case of, you know, this has happened, it’s now time for you to go (P21, menopause, 45-54).*  *I’d been there for three hours pushing that and they could see that labour wasn’t progressing as it should. And they kept saying they were bringing a doctor and nobody came. And I said, you know, “I’m in agony, I’m in agony” and they just dismissed it. Uhm. And I did ask for a caesarean as well. I said “I just want, can you just do a caesarean please?” And they were like “no. It’s too late, it’s too late, it’s too late”, you know, and whether it was too late I don’t know. But there was another two hours before somebody came (P10, menopause, 45-54).*  *So I asked the midwife to break my waters and she did. So you know, I’ve always wondered, was that a big mistake, you know, did it make it, did it bring it on too fast? (P24, menopause, 65+)*  *Lots of people who have, and lots of my friends and stuff. I don’t think really understand what I’m on about because they just had tears or caesareans and their stitches (P2, post-natal, 35-44).*  *My priority would be awareness, awareness posters, leaflets, information even in the maternity packs when women first know they’re pregnant and they get these packs of the hospital. But I know it’s scary to to tell women these things can happen. They don’t know when the first time you’re having a baby they don’t wanna know these things can happen, but it’s there (P39, menopause, 45-54).* |
| **3.** **Continuity and timeliness of care**  **Inadequacy of the six-week check**    **Waiting lists and chasing services**  **Mental and emotional needs ignored** | *From my own experience either there should be a check at six weeks or even earlier, really four weeks, whatever by a GP who can signpost you to things. (P30, post-natal, 35-44).*  *You should still be checked. Like I said, I ended up with an infection and I I pushed for a six-week check because when I rang they were like oh, we don’t really recommend them anymore. I was like, well I would like one please (P36, post-natal, 25-34).*  *So even after the six-week check, nobody*  *checked me at that point (P9, post-natal, 25-*  *34).*  *because at every stage of my treatment, I have had to fight for it, literally. “I need to be referred for this. I, I, I want that,” and ringing up, and ringing up and saying, “What’s happening with this? (P15, post-natal, 25-34).*  *Yeah, I’ve chased and chased and chased with, not with my gynae, because they, you know, they, they’re doing, my surgeon wants another surgeon in with him, and he said, “I want her to examine you and see you, 'cause I want her in theatre with me, 'cause you’re a little bit complex,” and that was done and I, and I met her and she said, “You’ll need a scan,” so I’ve had the scan, “You need urodynamics,” I’ve had appointments, yeah, they were cancelled but I, they’ve been reinstated with new dates, it’s just the colorectal surgeon, I’ve chased and chased his secretary, “Oh, you’re, you’re on the waiting list, they’re dealing with the main terms of priorities,” and then another six months will go, “Oh, you’ll be dealt with eventually, the surgeon’s looking at the list, and you’ll be dealt as a priority,” and every time you hear that you think, “Well, am I not a priority? (P28, post-natal, 35-44).*  *So I went three years before I had my second, because I was just like, “I can’t deal with everything that was going on,” and I had a lot of trauma from that first birth that I, I needed to work through before I had my second (P15, post-natal, 25-34).*  *It’s also a little bit like you can’t, I’m not, not that*  *I, I don’t wanna kind of blame anyone, but I just*  *would like to know what happened to try and*  *process it. Yeah, you know, ‘cause there is*  *underlying trauma, mentally, I guess from*  *having to, you know, being, I was 32 when I had*  *my first and, and pretty much losing bodily*  *function. Gradually, and I’m only 41 now, and*  *not being able to kind of run like I used to love to.*  *And, you know, there’s, there’s a lot of …*  *changes that you have to adapt to mentally.*  *And it’s, it would be really nice to be able to, to*  *just know, and then you could process it and then*  *you could work through it, and then you could*  *put it, you know … you’d be at peace with it (P22,*  *menopause, 35-44).*  *I mean it’s affecting me mentally more than it is*  *physically to be honest (P35, menopause, 55-64).* |
