# Supplementary material for: Women’s experiences of anal incontinence following vaginal birth: A qualitative study of missed opportunities in routine care contacts
Source: PLoS One. 2023 Jun 27;18(6):e0287779. doi: 10.1371/journal.pone.0287779 (PMC10298771; doi:10.1371/journal.pone.0287779)
Supplement: Attachment — Submitted filename: Responses to reviewers comments final.docx [file pone.0287779.s002.docx]

Dear Editor,

Thank you for the opportunity to address reviewer comments on the manuscript titled ‘Women's experiences of anal incontinence following vaginal birth: a qualitative study of missed opportunities in routine care contacts’ (PONE-D-23-06127).

We have addressed each comment in turn, and describe below our response to each comment.

| **Comment from reviewer/ editor** | **Response** |
| --- | --- |
| Please ensure that your manuscript meets PLOS ONE's style requirements, including those for file naming. The PLOS ONE style templates can be found at | We have been through the guidelines for the manuscript and made changes to formatting accordingly. |
| In the ethics statement in the Methods, you have specified that verbal consent was obtained. Please provide additional details regarding how this consent was documented and witnessed, and state whether this was approved by the IRB | Consent was not documented, but interviews only proceeded if consent was obtained. Participants were given the option of providing verbal consent; audio recorded at the start of the interview and saved in a separate file on an encrypted university drive or written consent; returned to the research team by email and saved in a separate file to the interview file on an encrypted university drive.  All consent files were saved separately to the interview files and labelled with a unique participant ID to ensure anonymity. This process was in line with ethical approval gained for the study. |
| Thank you for stating the following in the Competing Interests section:  “I have read the Journal's policy and the authors of this manuscript have the following competing interests:  Professor Michael Keighley is Trustee and President of the MASIC Foundation but this has not impacted on conduct of study or publication, CEO of Keighleycolo Ltd involved with medical reports for court in which OASI patients have been seen but no conflict as this simply informed me of the consequences of the injury during one to one face to face consultations. Trustee Friends of Vellore UK also has helped to understand impact of OASI in the Asian community in India. Joint holder of two i4i NIHR grants for a device for the treatment of fistula in fact of value to some OASI patients.”  Please confirm that this does not alter your adherence to all PLOS ONE policies on sharing data and materials, by including the following statement: "This does not alter our adherence to PLOS ONE policies on sharing data and materials.” (as detailed online in our guide for authors http://journals.plos.org/plosone/s/competing-interests). If there are restrictions on sharing of data and/or materials, please state these. Please note that we cannot proceed with consideration of your article until this information has been declared.  Please include your updated Competing Interests statement in your cover letter; we will change the online submission form on your behalf. | Thank you for this comment.  In relation to the disclosed conflict of interest we confirm that this does not alter our adherence to PLOS ONE policies on sharing data and materials.  Professor Keighley has acted as an expert witness during his career, but at no point in the interviews were women asked about legal cases. Furthermore, Professor Keighley was not involved in interviewing women or recruiting women. There is no way of knowing if he acted as an expert witness for recruited women, but it would have had no impact on recruitment or data collection. |
| In your Data Availability statement, you have not specified where the minimal data set underlying the results described in your manuscript can be found. PLOS defines a study's minimal data set as the underlying data used to reach the conclusions drawn in the manuscript and any additional data required to replicate the reported study findings in their entirety. All PLOS journals require that the minimal data set be made fully available. For more information about our data policy, please see <http://journals.plos.org/plosone/s/data-availability>.  Upon re-submitting your revised manuscript, please upload your study’s minimal underlying data set as either Supporting Information files or to a stable, public repository and include the relevant URLs, DOIs, or accession numbers within your revised cover letter. For a list of acceptable repositories, please see http://journals.plos.org/plosone/s/data-availability#loc-recommended-repositories. Any potentially identifying patient information must be fully anonymized.  We will update your Data Availability statement to reflect the information you provide in your cover letter. | As this study used qualitative methods of data collection (interviews), there is no single data set, and replicability of analysis is not an appropriate consideration.  Guidelines for the data policy suggest that excerpts of the transcripts relevant to the study are made available in an appropriate data repository. We do not feel this is something that we are able to do for this study, based on the ethical approvals and consent obtained for this study. We have ethical approval and consent from participants to include quotes, but any more than that is not covered by ethical approval or consent. We also believe it would be inappropriate to include more than short quotes, as it increases the chances that data is identifiable and therefore does not fulfil anonymity requirements.  We therefore do not feel any additional data should be shared in a repository, but would be happy if readers want to contact the lead author to discuss any elements of the data. |
| Your ethics statement should only appear in the Methods section of your manuscript. If your ethics statement is written in any section besides the Methods, please move it to the Methods section and delete it from any other section. Please ensure that your ethics statement is included in your manuscript, as the ethics statement entered into the online submission form will not be published alongside your manuscript. | We have moved the ethics statement to the methods section of the manuscript in line with this comments. It has been deleted from its original position. |
| Please review your reference list to ensure that it is complete and correct. If you have cited papers that have been retracted, please include the rationale for doing so in the manuscript text, or remove these references and replace them with relevant current references. Any changes to the reference list should be mentioned in the rebuttal letter that accompanies your revised manuscript. If you need to cite a retracted article, indicate the article’s retracted status in the References list and also include a citation and full reference for the retraction notice. | We have checked over the reference list and are not aware of any papers that have been cited in the manuscript. |
| Reviewer #1: Overall a well-structured, appropriately designed and original piece of research. Importantly, the value of this research in terms of the existing issue are set out clearly and potential avenues for improving experiences of women after childbirth injury are suggested without breaching the capabilities of qualitative research. An overview of the themes and subgroups in each would add clarity before the main text of the results. | Thank you for the positive comments about the manuscript.  We have added to the overview of the themes at the start of the results section, by adding in the subthemes for each theme. |
| Reviewer #2: Abstract P2  Clear, focused, check for unnecessary capitalisation in the results section | We have checked the results section of the abstract, and removed any unnecessary capitalisation. |
| Intro P3  Line 54 ‘nulliparous women’, could the authors add a brief explanation of this term please, | We have included a definition of what we mean by ‘Nulliparous women’ as ‘women who have never had a live birth.’ |
| Line 55 ‘OASI injury’ could the authors add a brief explanation of this term please | ‘OASI injury’ is written in full in line 53, but we have added a brief description that is designed to compliment the explanation in the previous paragraph. |
| Line 56 ‘Women of Indian and Pakistani origin are significantly more likely to sustain an OASI during childbirth (13,14, 58 15).’ Could you say briefly why this is please | The reasons behind this risk factor are likely to be complex and multifactorial. We have added a sentence to explain this, and an appropriate reference. |
| Line 61, could you add a sentence adding the implications please. | We have added a line to explain that the increase in risk factors may have likely attributed to the rise in incidence |
| Lin 67, could you add a sentence to say why this is the case please, it’s a fascinating study, and not everyone will be a medical professional, or familiar with the field. | We have added a short sentence to attempt to explain why urinary incontinence is discussed more than AI. |
| Methods P4  P4 Line 73 date needed please | We have included the date of the Ockenden review. |
| P5 line 99 ‘Participants were also recruited via five hospitals across the UK’ could you say which countries, regions please | We have added in the five regions of hospitals that participants were recruited from |
| P5 line . ‘Aiming for a maximum variation sample, women were recruited with varied backgrounds according to ethnicity, deprivation’ could you say why you wanted a variation sample, and what type of deprivation/how you were assessing deprivation please, | We have explained why we were aiming for a variation within the sample, and have explained how we assessed deprivation. |
| P5  Line 110 the PPI section is interesting, could you provide a sentence or detail about how patients were involved, and what influence this had at each level of involvement please. | We have added to this section to show the different points PPI was used, and the benefits it had on the study. |
| There is no mention of gaining ethical approval for the study, or the approval number in the methods section, I'm sure its an oversight, could you add it please. | Thank you for highlighting this. In line with the Editor’s comment we have moved the Ethical Statement into the Methods section rather than at the end of the manuscript. |
| P6 Data analysis  Whilst the whole paper is very well written and coherent, there is not enough detail or transparency about how the stages of analysis were completed, and the results were arrived at, this needs to be more explicitly included. | The data analysis followed the well-established method of Thematic Analysis, outlined by the work of Braun and Clarke. This follows six steps which have been detailed in the paper. We have added to this section to make it clearer and provided information for each step of the process. |
| Results P7  P8 Table 1 What type of deprivation is being measured here, could you add more detail to the axes please | We have added information as a note to the table to describe how deprivation was measured. |
| There is some unnecessary capitalisation mid-sentence throughout this section. | We have checked the results section for unnecessary capitalisation and have corrected this. |
| Its only a small study, could the authors add in brackets where they say many participants, how many, to give an idea of context. | We have considered this point, but feel that as this is a qualitative study it is not usual to quantify findings in this way. The use of ‘many’ or similar was intended to imply that it was a strong feeling amongst the participants with similar responses. Further, we feel a sample size of 41 is a moderate size for a qualitative study. |
| This whole sub-section is very well written, with clear explanation, but at the moment it is not clear where these themes came from, this needs more explanation and linkage in the Methods section, good illustrative quotes. | We have added some further explanation to how the themes were developed. We hope this will address this concern. |
| Discussion P20  All the comments are accurate and relate to the paper, but it feels more like a summary rather than key points arising, and their implications,  what are the competing tensions? and how these concerns might be addressed - in several areas, e.g. societally, patient information, pregnancy related services, knowledge of health professionals… policies and publicity…where are the blockages, what needs to happen for services to communicate better… I'm not saying you need to resolve everything, but going beyond, to give specific ideas, pointers for better, more joined up practice, information for women ect, would lead to a stronger discussion section  what does it add, what needs to happen, what are the implications? | We have tightened up the implications for practice and research, and added some information about possible solutions to services. |
| Conclusion P23  A really clear paper, with quality data, it would benefit from a stronger, bolder conclusion, | We have reworked the conclusion section to tighten some of the messages. |
| Reviewer #3: I read with great interest the Manuscript titled “Women's experiences of anal incontinence following vaginal birth: a qualitative study of missed opportunities in routine care contacts” (PONE-D-23-06127), which falls within the aim of this Journal.  In my honest opinion, the topic is interesting enough to attract the readers’ attention. Methodology is accurate and conclusions are supported by the data analysis. Nevertheless, authors should clarify some point and improve the discussion citing relevant and novel key articles about the topic. | Thank you for the positive comments about the manuscript.  We have revisited the discussion section (particularly the section about comparison with previous literature). We are confident that this section presents relevant and novel literature on the topic to compliment what is presented in the introduction section. We have also tightened up the strengths and limitations section which hopefully helps to clarify this section. |
| Authors should consider the following recommendations:  - Inclusion/exclusion criteria should be better clarified. | We have amended this section to show inclusion and exclusion criteria as a list to make it easier for readers. |
| The Authors did not mention the sample size calculation for their study. It is essential to specify this data in order to guarantee an adequate significance of the results obtained by the Authors. | As this is a qualitative study we did not conduct a sample size calculation. This is not part of qualitative methodology. In qualitative research, sample sizes are determined by data saturation; a sufficient number of interviews have been conducted whereby the research question is sufficiently answered, no further new information is evident, and no new themes are identified. |
| The authors have not adequately highlighted the strengths and limitations of their study. | We have reviewed the ‘Strengths and Weaknesses’ section of the manuscript and have further emphasised the strengths and limitations of the study. |
| What are the actual clinical implications of this study? it is important to report the results obtained by the authors in the context of clinical practice and to adequately highlight what contribution this study adds to the literature already existing on the topic and to future study perspectives | As with the earlier comment we have added to the implications section of the paper. |
| Does this manuscript conform the Enhancing the QUAlity and Transparency Of health Research (EQUATOR) network guidelines? It would be mandatory to declare about this element. | In the methods we have noted that the Standards for Reporting Qualitative Research framework was used for reporting the research. The SRQR is listed on the EQUATOR network as one of the appropriate guidelines for qualitative research. |
| Authors should add further details to discuss the role of the perineum protection techniques during the management of the second phase of labour and the effect on the postpartum period (authors may refer to: PMID: 25909491; PMID: 24942141). | We have added in a statement about perineal protection techniques in the introduction. We did not include it initially because the focus of this study is on the impact of the injury rather than the prevention of it. Thank you for the useful references. We have included one of them. |
